# Hashimoto Encephalopathy of a Middle-Aged Man With Progressive Symptoms of Dementia

**DOI:** 10.7759/cureus.27518

**Published:** 2022-07-31

**Authors:** Noritaka Katagiri, Ryuichi Ohta, Fumiko Yamane, Chiaki Sano

**Affiliations:** 1 Family Medicine, Department of Community Medicine Management, Faculty of Medicine, Shimane University, Izumo, JPN; 2 Community Care, Unnan City Hospital, Unnan, JPN; 3 Department of Community Medicine Management, Faculty of Medicine, Shimane University, Izumo, JPN

**Keywords:** general physician, rural hospital, subacute cognitive impairment, antithyroid antibody, autoimmune encephalitis, hashimoto’s encephalopathy

## Abstract

Autoimmune encephalitis is caused by immunological reactions showing unconsciousness, agitation, and other neurological symptoms. Autoimmune diseases can be related to autoantibodies, causing encephalitis. These autoantibody-related encephalitides could appear in various clinical courses. As laboratory tests for detecting these antibodies are limited, diagnosis is difficult. Hashimoto’s encephalopathy is autoimmune encephalitis caused by antibodies against the thyroid gland. This time, we experienced a case of a 69-year-old man with a chief complaint of subacute progression of amnesia and suspected autoimmune encephalitis, who was finally diagnosed with Hashimoto’s encephalopathy in a rural community hospital. In this case, clinicians should consider Hashimoto's encephalopathy as a differential diagnosis and measure antithyroid antibodies when acute or subacute onset cognitive impairment is observed in middle-aged patients. As a super-aging society significantly affects community hospitals, general physicians need to start treatments for encephalopathy and encephalitis when clinicians suspect the disease and rule out other critical diseases.

## Introduction

Autoimmune encephalitis is caused by various immunological reactions in the brain manifesting as various symptoms [1]. Encephalitis exhibits unconsciousness, agitation, and other neurological symptoms depending on the extent of inflammation of the brain. The etiologies vary owing to various pathophysiology that causes inflammation in the brain. Autoimmune diseases, such as systemic lupus erythematosus, Behçet's disease, and other connective tissue diseases related to autoantibodies, cause encephalitis [2-4]. Limbic encephalitis is also a form of autoimmune encephalitis and is associated with antibodies, such as Hu, Ma2, anti-glutamic acid decarboxylase, anti-N-methyl-d-aspartic acid receptor, anti-N-methyl-d-aspartic acid receptor, anti-leucine-rich glioma-inactivated 1, and anti-contactin-associated protein-like 2 have been reported previously [5,6]. Autoantibody-related encephalitis could appear in various clinical courses. Laboratory tests for detecting these antibodies are limited, even in developed countries, making diagnosis difficult. Hashimoto’s encephalopathy is autoimmune encephalitis caused by the thyroid's antibodies [7]. Hashimoto’s encephalopathy is not directly related to Hashimoto’s thyroiditis because many patients with Hashimoto’s encephalopathy do not have abnormalities in thyroid function [7]. Treatments for encephalopathy and encephalitis may start when clinicians suspect the disease and rule out other critical diseases.

The frequency of the diagnosis of autoimmune encephalitis may be increasing because of advancements in the recognition and understanding of the disease [8]. However, practical approaches can be challenging in rural contexts. The limitations of the diagnosis may be due to the lack of healthcare resources and limitations in various clinical tests of autoantibodies. Here we report a middle-aged patient with chief complaints of subacute progression of amnesia and suspected autoimmune encephalitis who was finally diagnosed with Hashimoto’s encephalopathy in a rural community hospital. We also describe the precise clinical course and discuss the challenges and practical approaches to the diagnosis of Hashimoto’s encephalopathy in rural contexts.

## Case presentation

A 69-year-old man presented to our hospital with complaints of one month of altered consciousness and difficulty walking by himself. He visited his local doctor 13 days before admission and was diagnosed with Alzheimer's and vascular dementia with an HDS-R (Hasegawa Dementia Scale-Revised) score of 19. One week before admission, he could speak and walk; however, on the day before admission, he began to have visual hallucinations and developed insomnia. On the day of admission, he was unable to speak or walk, and his room was in disarray. He had a history of Alzheimer's disease and vascular dementia, infarction of the left lenticular striate artery region, and right-hand dexterity disorder (six years ago); osteoarthritis of the lumbar spine (two years ago); and scalp laceration (seven years ago). His drug history included tamsulosin, mirabegron, and clopidogrel use. He had a history of episodes of dangerous driving and getting lost three months before his admission.

His vital signs were a blood pressure of 128/64 mmHg, a body temperature of 37.5°C, pulse rate of 78 beats/min, respiratory rate of 18 breaths/min, and SpO2 of 98%. He did not follow physicians’ orders. His physical examination revealed poor oral hygiene and stiffness of all extremities. The other neurological examinations showed no specific abnormalities regarding the central nervous system. There were also no abnormalities of heart, lung, or dermal findings. Laboratory findings included blood tests with a total bilirubin of 1.7 mg/dL, direct bilirubin of 0.5 mg/dL, creatinine kinase of 1523 U/L, vitamin B1 of 32.0 ng/mL, C-reactive protein of 0.14 mg/dL, erythrocyte sedimentation rate of 6 mm, and urine test with ketone body 3+ (Table 1).

**Table 1:** Initial laboratory data of the patient

Marker	Level	Reference
White blood cells	6.4	3.5–9.1 × 10^3^/μL
Neutrophils	69.4	44.0%–72.0%
Lymphocytes	18.1	18.0%–59.0%
Monocytes	11.7	0.0%–12.0%
Eosinophils	0.2	0.0%–10.0%
Basophils	0.6	0.0%–3.0%
Red blood cells	4.29	3.76–5.50 × 10^6^/μL
Hemoglobin	14.5	11.3–15.2 g/dL
Hematocrit	43.3	33.4%–44.9%
Mean corpuscular volume	101	79.0–100.0 fl
Platelets	18.4	13.0–36.9 × 10^4^/μL
Erythrocyte sedimentation rate	6	2–10 mm/h
Total protein	7.7	6.5–8.3 g/dL
Albumin	4.6	3.8–5.3 g/dL
Total bilirubin	1.7	0.2–1.2 mg/dL
Direct bilirubin	0.5	0–0.4 mg/dL
Aspartate aminotransferase	71	8–38 IU/L
Alanine aminotransferase	28	4–43 IU/L
Alkaline phosphatase	92	106–322 U/L
γ-Glutamyl transpeptidase	14	<48 IU/L
Lactate dehydrogenase	260	121–245 U/L
Uric acid	7.6	3.0–6.9 mg/dL
Blood urea nitrogen	36.3	8–20 mg/dL
Creatinine	0.85	0.40–1.10 mg/dL
eGFR	68.7	>60.0 mL/min/L
Serum Na	142	135–150 mEq/L
Serum K	4.1	3.5–5.3 mEq/L
Serum Cl	103	98–110 mEq/L
Serum Ca	9.9	3.5–5.3 mg/dL
Serum P	3.3	0.2–1.2 mg/dL
Serum Mg	2.1	1.8–2.3 mg/dL
CK	1523	56–244 U/L
CRP	0.14	<0.30 mg/dL
TSH	0.54	0.35–4.94 μIU/mL
Free T4	1	0.70–1.48 ng/dL
Vitamin B1	32	21.3–81.9 pg/mL
Folic acid	5.7	>4.0 ng/mL
Urine test		
Leukocyte	-	
Nitrite	-	
Protein	1+	
Glucose	-	
Urobilinogen	1+	
Bilirubin	-	
Ketone	3+	
Blood	1+	
pH	6	
Specific gravity	1.03	

**Figure 1:**
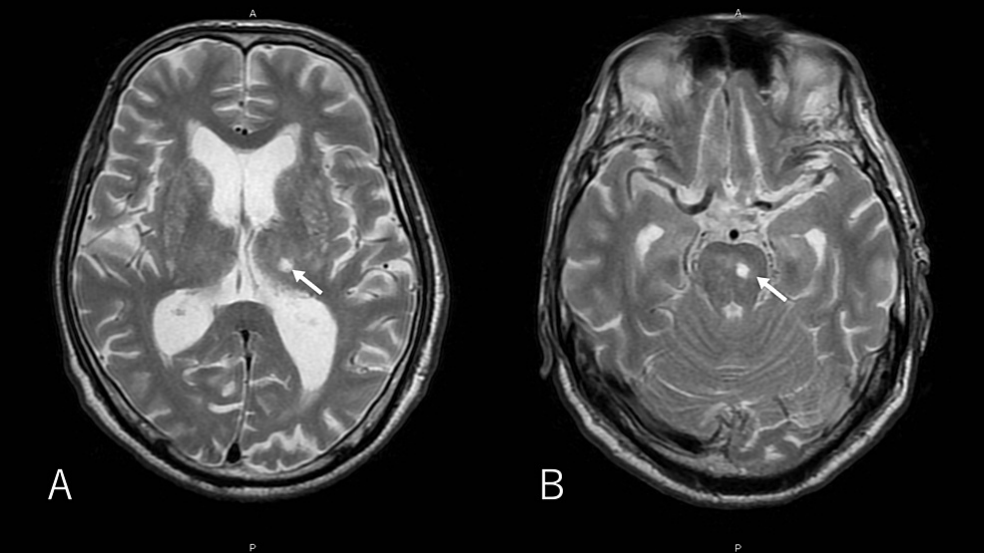
Magnetic Resonance Imaging (T2WI sequence) on the third day of hospitalization showing high signal in the left thalamus (A) and left side of the pons (B).

Head CT showed no intracranial hematoma, blood lesions, or masses and no obvious ventricular enlargement. Meanwhile, brain MRI showed a high signal intensity on the T2WI sequence and low signal intensity on the FLAIR sequence in the left thalamus and left side of the pons, indicating chronic cerebral infarction (Figure 1). 

**Figure 2:**
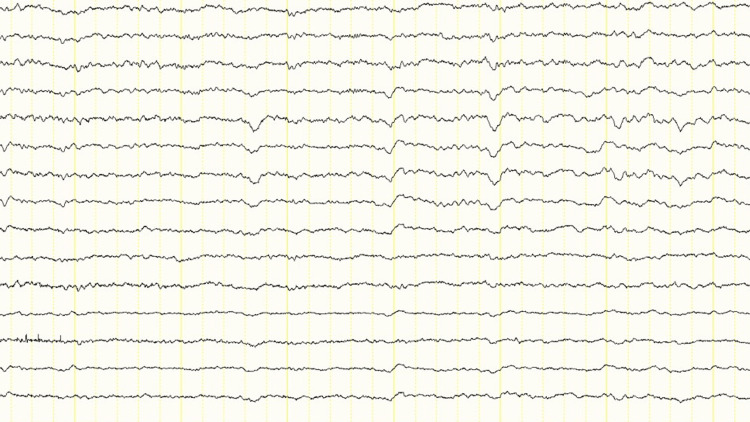
The electroencephalogram showing slow waves with no spikes

On the first day of hospitalization, vitamin B1 supplementation was administered to treat possible Wernicke's encephalopathy. On the third day of admission, CSF puncture revealed an elevated cell count of 7/µL (all the cells, lymphocytes) and a protein level of 65 mg/dL (normal range, under 45), and the patient was started on acyclovir with a diagnosis of herpes encephalitis. An electroencephalogram was performed to differentiate non-convulsive epileptic seizures from prolonged loss of consciousness. The electroencephalogram showed slow waves with no spikes (Figure 2).

On the fifth day of admission, there was no change in consciousness; considering the possibility of autoimmune encephalitis, intravenous methylprednisolone (1000 mg) was administered for three days. The dose was then reduced to 10 mg per week.

Additional blood samples before the intravenous methylprednisolone were also revealed to be positive for anti-thyroglobulin antibodies and negative for anti-thyroid peroxidase antibody, screening of antinuclear antibodies (homogenous, nucleolar, peripheral, discrete, and cytoplasm), and Anti-N-methyl-d-aspartate (NMDA) receptor antibody which was checked with the support of the local university. As the screening of the antinuclear tests was negative, we did not check specific autoimmune antibodies related to autoimmune diseases. The diagnosis of Hashimoto's encephalopathy was first considered due to the acute and progressive nature of the disease. Based on the diagnosis, the acyclovir infusion was terminated. The patient was treated with a reduced dose of prednisolone with no apparent worsening of consciousness. The patient's progress was good, and he was scheduled to be transferred to the rehabilitation ward for discharge to his home.

## Discussion

In this case, a middle-aged man with subacute onset of consciousness impairment was diagnosed with Hashimoto's encephalopathy, and his symptoms of consciousness impairment and cognitive decline were successfully treated with pulse steroid therapy. The diagnosis of Hashimoto's encephalopathy was made based on the improved course of the disease caused by the administration of steroids and the positive test results for anti-thyroglobulin antibodies. Although, due to the variety of clinical manifestations, it is difficult to diagnose Hashimoto's encephalopathy based on clinical symptoms alone because of the variety of clinical manifestations [9,10], early treatment of acute cognitive decline can be initiated by measuring anti-thyroglobulin antibodies with consideration for Hashimoto's encephalopathy.

Autoimmune encephalitis is a differential diagnosis of acute and subacute CNS symptoms. It should be included in the diagnosis of sudden cognitive decline and impaired consciousness [6]. Autoimmune encephalitis is associated with the presence of numerous autoantibodies, and measurement of these antibodies may lead to the diagnosis. However, Japan's insurance does not cover antibody tests for autoimmune encephalitis; only anti-thyroid and antinuclear antibodies can be checked. Hu, Ma2, anti-glutamic acid decarboxylase, anti-N-methyl-d-aspartic acid receptor, anti-N-methyl-d-aspartic acid receptor, anti-leucine-rich glioma-inactivated 1, and anti-contactin-associated protein-like 2 cannot be checked without medical universities and private companies support. There is little need to wait for test results because it takes time to obtain results, and there are many antibody-negative cases [11]. Anti-thyroid antibodies should be measured in middle-aged patients with acute or subacute cognitive decline with consideration for Hashimoto's encephalopathy.

Hashimoto's encephalopathy is a treatable disorder that responds well to steroids [12], but a follow-up period is required until symptoms resolve. In general, 93% of patients with Hashimoto's encephalopathy show complete or partial remission within three months of diagnosis [10]. On the other hand, a delay in diagnosis can lead to cognitive decline in 25% of patients [13], which may result in irreversible central nervous system damage [14]. Hashimoto's encephalopathy should always be considered as a differential diagnosis in disorders of consciousness because of the variety and often overlooked symptoms of Hashimoto's encephalopathy and the possibility of after-effects due to the months-long treatment process and delayed diagnosis. In our case, we cannot rule out other autoimmune disorders due to the lack of testing; hence, Hashimoto's encephalopathy cannot be definitively confirmed. In diagnosing and treating Hashimoto's encephalopathy, clinicians should consider not only quick treatments of this disease but also the possibility of other autoimmune encephalopathies because of the test limitation in rural contexts. 

Hashimoto's encephalopathy, autoimmune encephalitis specifically diagnosed in community hospitals, is likely to increase in an aging society in the future; therefore, general physicians must diagnose it appropriately. To facilitate the diagnosis and treatment of Hashimoto's encephalopathy in community hospitals, it is necessary to consider systemic autoimmune diseases such as systemic lupus erythematosus, Behçet's disease, and herpetic encephalitis for acute and subacute cognitive decline in middle-aged patients [15]. However, since antibodies are not covered by insurance, we believe that clinical diagnosis of autoimmune encephalitis, initiation of steroid therapy without waiting for antibody testing, and concomitant testing for anti-thyroid antibodies, taking into account Hashimoto's encephalopathy, will improve patient prognosis. In aging societies, elderly patients with vague symptoms, including gradual cognitive decline, may manage their symptoms through self-management and limited social care in rural contexts [16,17]. To facilitate effective diagnosis of autoimmune diseases in rural contexts, inter- and trans-professional education is needed so that older people can effectively approach medical care [18,19].

## Conclusions

We reported a case of Hashimoto's encephalopathy with a broad spectrum of symptoms, showing the difficulty of diagnosing autoimmune encephalitis based on the clinical course alone. Hashimoto's encephalopathy should be considered when acute or subacute onset cognitive impairment is observed in middle-aged patients. A super-aging society significantly affects community hospitals because there are many elderly patients with unconsciousness from various medical reasons. General physicians must consider Hashimoto's encephalopathy for its prompt diagnosis and treatment.
